# Doping Strontium into Neodymium Manganites Nanocomposites for Enhanced Visible light Driven Photocatalysis

**DOI:** 10.1038/s41598-019-50393-9

**Published:** 2019-09-26

**Authors:** I. A. Abdel-Latif, L. A. Al-Hajji, M. Faisal, Adel A. Ismail

**Affiliations:** 1 Physics Department, College of Science, Najran University, Najran, P.O. Box 1988, Najran, 11001 Saudi Arabia; 2 Nanotechnologyand and Advanced Materials Program, Energy & Building Research Center, Kuwait Institute for Scientific Research (KISR), P.O. Box 24885, Safat, 13109 Kuwait; 3 Advanced Materials and Nano-Research Centre, Najran University, P.O. Box: 1988, Najran, 11001 Saudi Arabia; 4 Reactor Physics Department, NRC, Atomic Energy Authority, Abou Zabaal P.O. 13759, Cairo, Egypt; 5 Central Metallurgical R& D Institute, CMRDI, Helwan, Cairo, Egypt

**Keywords:** Chemistry, Nanoscience and technology

## Abstract

Nd_1−x_Sr_x_MnO_3_ nanocomposites perovskites were synthesized using sol gel method at different Sr content x = 0.3, 0.5, 0.7, and 0.9. The photocatalytic performance of the Nd_1−x_Sr_x_MnO_3_ nanocomposites for photodegradation of Acridine orange dye (AO) was evaluated over visible light illumination. The single phase of orthorhombic pbnm was formed for x = 0.3 and 0.5; however monoclinic and orthorhombic were observed at x = 0.7 and 0.9. The Energy gap of the Nd_1−x_Sr_x_MnO_3_ nanocomposites were estimated for all concentrations to be in the range of 3 ± 0.05 eV. The photocatalytic efficiency of Nd_0.3_Sr_0.7_MnO_3_ nanocomposite was 95% of the initial AO dye concentration within 3 h illumination time. The linear increase of the photodegradation rate was found in our samples as a result of the increase of Sr contents from 0.3 to 0.7wt %. Interestingly, the Nd_0.3_Sr_0.7_MnO_3_ content has the highest degradation rate of AO which is two times faster than undoped NdMnO_3_. This superior behavior in photocatalytic activity of Nd_0.3_Sr_0.7_MnO_3_ nanocomposite emerges from large surface area, structural anisotropy, and small particle size. These findings shows convincingly that the Nd_1−x_Sr_x_MnO_3_ photocatalysts possess great promise for visible light driven photodegradation of AO dye.

## Introduction

Serious environmental problems and daily energy demand are crucial subject to which increase the scientific attention in order to develop new nontoxic, inexpensive, stable, and efficient materials to solve these issues. Photocatalysis studies are grown up as an important in the field of clean energy applications. Rare earth perovskites ABO_3_ are used recently as photocatalysis^1–5^ and these class of materials are key type of oxides due to their unusual physical and chemical properties^6–10^. The effect of nanocrystalline size is an efficient parameter that plays key role in these physical and the chemical properties showed by such materials^11–14^. The position of oxygen atoms around both transition metal cations A and rare earth cations B in the ABO_3_ perovskites reflects how its importance role in determination its exciting properties and thus its potential applications. The transfer of electrons between atoms that occupy B-site is not directly transfer between these atoms but through oxygen atoms forming octahedral surround the transition metal atoms in B-site. From the other side the distortion of the octahedral site in the perovskite may change the electronic and magnetic properties that reflects the importance of the corner-shared octahedral BO_6_ lattice site in these materials^1^. Moreover, one parameter that affect activity in such materials is the mixed valence states of the transition metal at B-site. There are different methods in synthesis of rare earth manganites within perovskite-like structure such as solid state reaction^15–18^, chemical co-precipitation^19–21^, sol-gel^22–25^. The methods of synthesis play an important role in the formation of the required crystal structure and how the oxygen atoms in the octahedral are distributed beside the crystalline size. A lot of research work has been devoted to study photocatalysis performance of perovskite-like materials such as tantalate^26–29^, titanate^30–33^, ferrite^15–17^, vanadium and niobium^34–37^, and manganites^11–13^. These materials have displayed visible light photocatalytic activity because they have exclusive electronic properties, which correlated with their rich crystal structures^38–42^. The optimized band gap energy in such oxides, the doped divalent element rare-earth transition metal oxides of the perovskite-like structure, explains and enhances photogeneration of both electrons and holes and hence the separation of charge carriers^43^. More efforts and intensive research have been done and still now we need more activities in order to tuning their optical and electrical properties of these materials that may help us to control of their rational design structure by the cationic replacements in ABO_3_ pervoskite^3^. The promising photocatalytic performance of the perovskite compounds are observed as a result adapting their bandgap values to the produced visible-light absorption as well as the potentials of band edge to tailor the requirements of particular photocatalysis. Moreover, the lattice distortion presented in such materials correlated to the separation of charge carriers generated by photons^44,45^. The resulting distortion occurred in the bond angles between metal-ligand and the metal-ligand-metal is significantly affected their charge carriers as well as the band gap values^1,46,47^. The following parameters; the surface area, the phase structure, the size, and the crystallinity affect the efficiency of photocatalysts. Consequently, control of the size and the crystal phase as well as the shape of perovskites is fundamental and key parameter for estimating their phase-dependent photoactivity. Nd_0.6_Sr_0.4_MnO_3_ was examined as photocatalyst by Abdel-Latif *et al*.^1^, under visible light with different adjustment of perovskite to get highly produced photons and increasing both of the migration and separation of the photogenerated charge carriers over the photocatalytic reaction. Nd_0.6_Sr_0.4_MnO_3_ is a narrow band gap semiconductor material (with energy values ranged from 2–2.98 eV), which could be controlled by changing its annealing temperatures. In this work, the effect of strontium doping on the crystal structure and photocatalytic performance of neodymium manganites Nd_1−x_Sr_x_MnO_3_ (x = 0, 0.3, 0.5, 0.7, and 0.9) were studied in details. The linear increase in the photodegradation rate was found as a result of the increase in the Sr contents from 0.3 to 0.7wt %. Interestingly, the Nd_0.3_Sr_0.7_MnO_3_ content has the highest degradation rate of AO which is two times faster than undoped NdMnO_3_.

## Experimental Details

### Materials

Sr(NO_3_)_2_, Nd(NO_3_)_3_·6H_2_O, and Mn(NO_3_)_2_, citric acid, polyethylene glycol (average M.W. = 1900–2200), and NH_4_OH (28–30% NH_3_) are Sigma-Aldrich Chemicals and used without further purification.

### Preparation of Nd_1−x_Sr_x_MnO_3_ perovskites

Nd_1−x_Sr_x_MnO_3_ nanocomposites perovskites were synthesized using sol gel method. An appropriate amount of Nd(NO_3_)_3_·6H_2_O, Sr(NO_3_)_2_ and Mn(NO_3_)_2_·6H_2_O were dissolved in 100 ml H_2_O, and subsequently mixed with citric acid as chelating agent. The above mixed solution was stirring continuously for 1 h at 80 °C (the selected molar ratio of nitrate salts to citric acid in our case is 1:2). Then, 1 gm of polyethylene glycol agent was gradually added to the mixture as structure directing and the stirring was kept continuously for more two hours. The pH of the solution should be controlled to be 8 by adding few drops of NH_4_OH to produce pure solution. The produced solution was fired at 80 °C for 24 h to evaporate H_2_O and polymerization organic compounds including inorganic oxides until the formation of gels. The resulted as-prepared powder was fired at 500 °C for 6 hours to produce the required perovskite structure.

### Characterizations

Micrograph images were performed using Field emission-secondary electron microscope (FE-SEM) with a FE scanning electron microanalyzer (JEOL-6300F, 5 kV). XRD patterns were collected by PANalytical diffractometer using Cu X ray tube. BET surface areas of the prepared samples were measured at 77 K using a Quantachrome Autosorb 3B after the samples were vacuum-dried at 200 °C overnight. All the reflectance spectra for our samples were measured by UV-visible spectrophotometer (lambda 950 PerkinElmer) connected with universal reflectance accessory in the wavelength range from 200 to 800 nm. All spectra were collected at room-temperature. When the results of UV-vis diffuse reflectance spectra (*R*) were measured, they were converted to the Kubelka-Munk function *F(R)* to subtract the light absorption extent from scattering one.

### Photocatalysis experiments

In the photodegradation of Acridine orange dye (AO) reaction, AO was conducted as a probe pollutant to evaluate the photocatalytic performance of NdMnO_3_, Nd_0.7_Sr_0.3_MnO_3_, Nd_0.5_Sr_0.5_MnO_3_, Nd_0.3_Sr_0.7_MnO_3_ and Nd_0.1_Sr_0.9_MnO_3_ perovskites nanocomposites. 100 ml of AO dye [0.03 mM] and 0.5 gL^−1^ photocatalyst were mixed in photoreactor with magnetic stirring. The visible lamp (Osram, Germany) was horizontally fixed above the photoreactor with 10 cm distance. In dark, a suspension solution containing AO and nanocomposites was magnetically stirred for 2 h without illumination to obtain adsorption equilibrium. The adsorbed AO was taken into consideration through adsorption reactions. Throughout the experiment, the suspension was continuously purged with oxygen bubbling.

The photocatalytic activity of the catalysts for AO dye photodegradation was determined by recording the absorption spectra using UV-visible spectrophotometer at λ = 490 nm at different illumination time. The AO concentration was recorded absorbance matching to the maximum absorption wavelength of AO. The photocatalytic efficiency (PE%) was determined by employing the following equation:

$$PE\, \% =[\frac{({{\rm{C}}}_{{\rm{o}}}-{{\rm{C}}}_{{\rm{t}}})}{{{\rm{C}}}_{{\rm{o}}}}]\times 100 \% $$

where C_o_ and C_t_ are the initial AO dye concentrations before illumination, and the AO dye concentration at interval illumination time, respectively.

## Results and Discussions

### Crystal Structure Nd_1−x_Sr_x_MnO_3_

**Figure 1:**
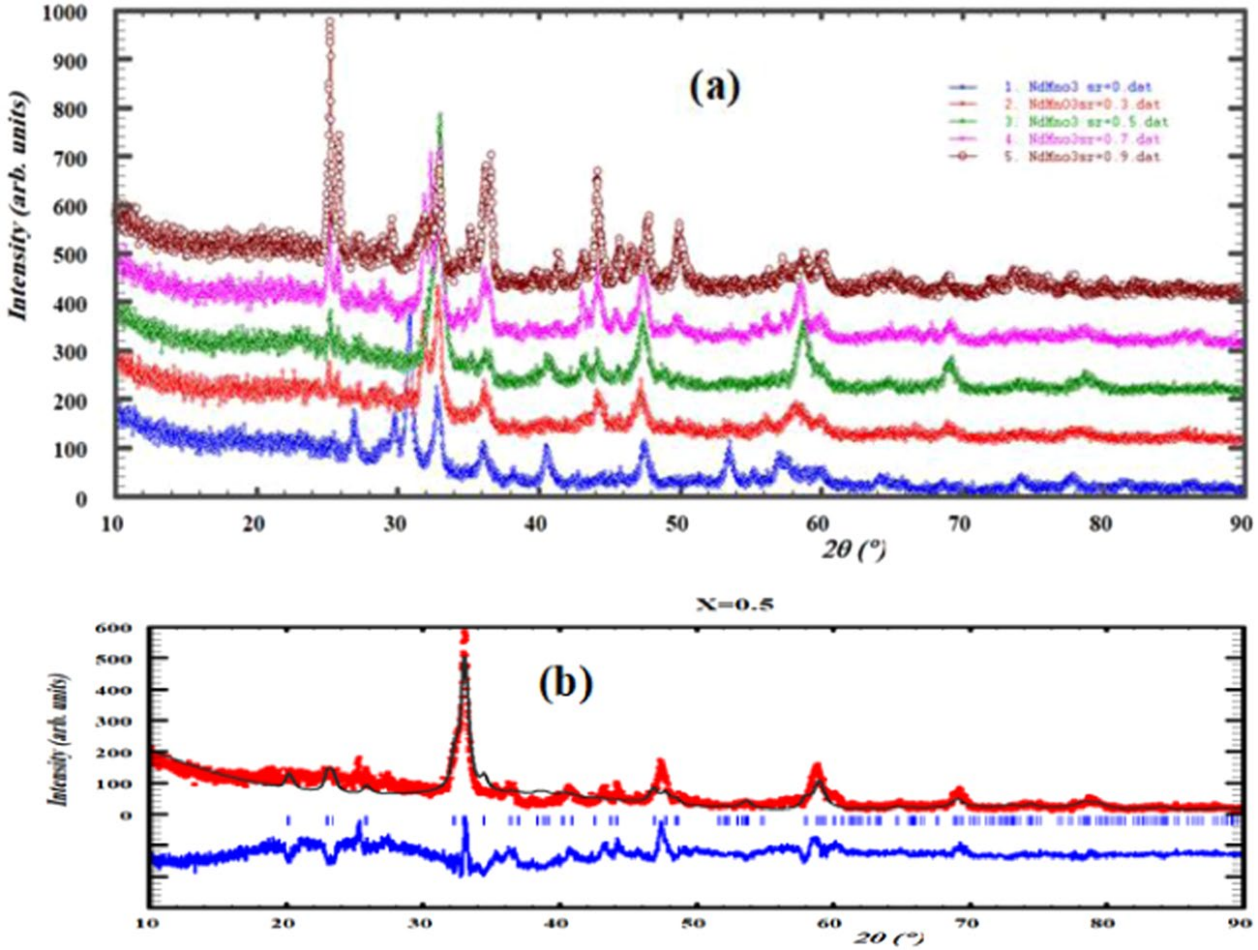
(**a**) XRD patterns of Sr_x_Nd_1−x_MnO_3_ (x = 0, 0.3, 0.5, 0.7, and 0.9). (**b**) XRD pattern of Sr_0.5_Nd_0.5_MnO_3_ and the Rietveld refinement using Fullprof software.

**Figure 2:**
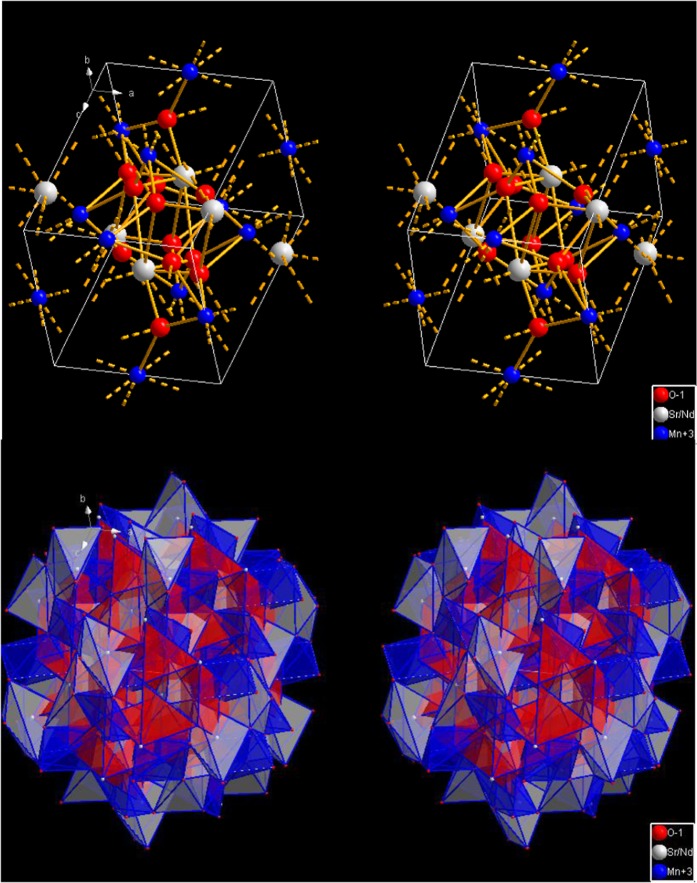
Unit cell representation of orthorhombic phase of Nd_0.5_Sr_0.5_MnO_3_ in 3-dimensions.

**Figure 3:**
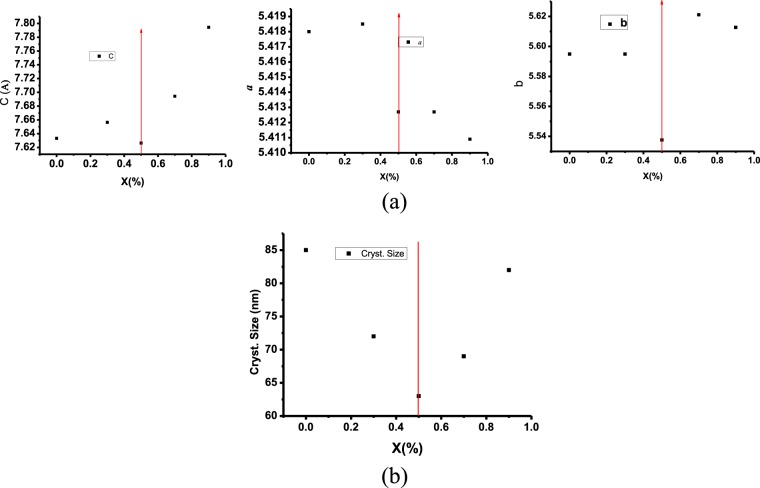
(**a**) Lattice parameters of the orthorhombic crystal system (**a**–**c**) as a function of Sr concentrations and (**b**) crystalline size as a function of Sr concentrations

The rare earth manganites is formed in the well-known perovskites crystal structure where they does not crystalize only in the cubic crystal system but also in the form of the orthorhombic, rhombohedral, hexagonal or monoclinic depends on the synthesis procedure and heat process. The different crystal structure systems of rare earth manganites were reported in different work, where they could be formed as cubic, orthorhombic, rhombohedral, hexagonal or monoclinic^1,3,9–16^. In this work, the effect^48^.

of strontium doping on the crystal structure is investigated in details using X-ray diffraction and the XRD patterns are shown Fig. 1a. Fitting of all the measured XRD patterns is carried out based on the Rietveld refinement using Fullprof software^44^ as shown in Fig. 1b. The obtained results from the refinements of all concentrations of Sr_x_Nd_1−x_MnO_3_ are listed in Table 1. The unit cell representation in 3-dimensions of the Nd_0.5_Sr_0.5_MnO_3_ that has the orthorhombic phase is shown in Fig. 2. The sample without strontium (x = 0) is found to have mixed crystal structure; monoclinic crystal system with 87% space group C2/c and 13% orthorhombic crystal system of space group Pbnm. As a result of adding strontium the monoclinic phase is completely transformed into the orthorhombic crystal structure form at x = 0.3 and x = 0.5. with increasing of the concentration of strontium more than 0.5 a new phase appeared (monoclinic with space group P21/n) along with the orthorhombic Pbnm phase. From the lattice parameters and crystalline size, as shown in Fig. 3, one can say that the concentration x = 0.5 is a transition point where the crystalline size decrease up till x = 0.5 then increase again.

**Table 1:** Crystal structure parameters and Energy gap of Sr_x_Nd_1−x_MnO_3_.

X	Crystal system	*a (Å)*	*b(Å)*	*c(Å)*	α	β	γ	Crystalline size (nm)	Energy gap (eV)
0	Ortho P b n m 13%	5.418(4)	5.630(4)	7.633(5)	90	90	90	85	3.046
	Mono C 2/c 87%	12.9900	10.2503	11.2042	90	128.23	90		
0.3	Ortho P b n m	5.4185	5.5949	7.6564	90	90	90	72	3.042
0.5	Ortho P b n m	5.4127	5.5376	7.6263	90	90	90	63	3.040
0.7	Ortho P b n m 26.7%	5.4127	5.6211	7.6943	90	90	90	69	3.00
	Mono P 21/n 73.3%	5.4632	5.6320	7.6404	90	89.82	90		
0.9	Ortho P b n m 4%	5.4109	5.6127	7.7943	90	90	90	82	3.02
	Monoclinic P 21/n 96%	5.4407	5.6067	7.614769	90	89.184	90

Electron density ρ(r) that scattered from the unit cell was calculated using Fourier method by subprogram in Full Prof software regardless of the symmetry. A Fast Fourier Transform (FFT) is applied in our case as a subroutine to calculate electron density according to the following expression^49^:

$$\rho (r)=\frac{1}{V}\sum _{{\boldsymbol{H}}}F({\boldsymbol{H}})exp\{-2\pi i({\boldsymbol{H}}\cdot {\boldsymbol{r}})\}$$

**Figure 4:**
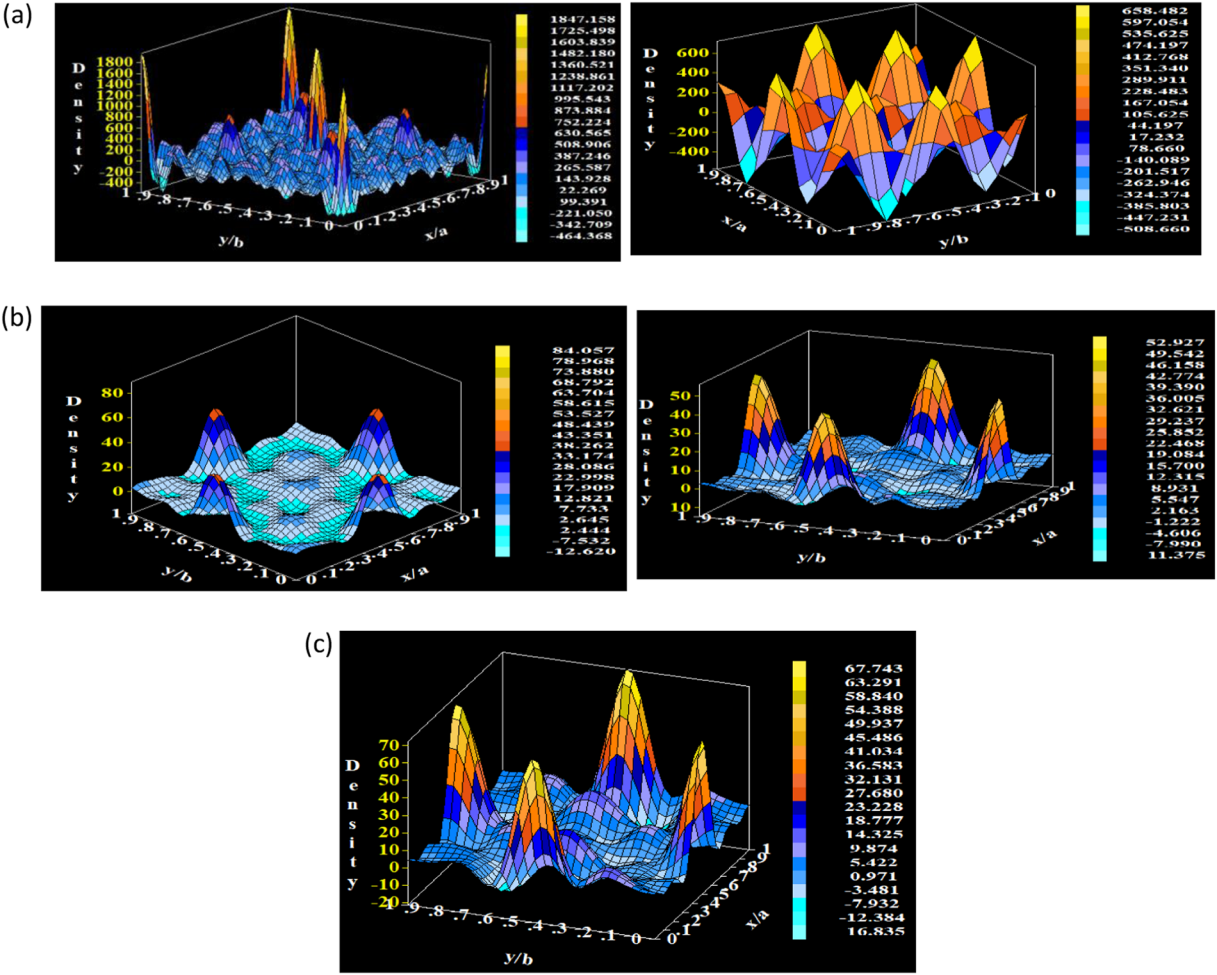
Electron density maps of Sr_x_Nd_1−x_MnO_3_ (x = 0, 0.3, 0.5, 0.7, and 0.9).

From this equation, it is clear that ρ(r) is function of the following parameters; the volume of the unit cell (V), the reciprocal lattice vector (H), the vector position inside the unit cell (r), and the complex Fourier coefficients F(H), which are applied to implement different types of Fourier syntheses. The units of ρ (r) are those of F(H) divided by those of V. The calculations of the density of electrons inside the unit cell for all the x concentrations of Nd_1−x_Sr_x_MnO_3_ are shown in Fig. 4. One can note from the electron density maps that the density of electrons for first two concentration x = 0 and x = 0.3 are comparatively higher than the other concentrations.

### Microstructure analysis

**Figure 5:**
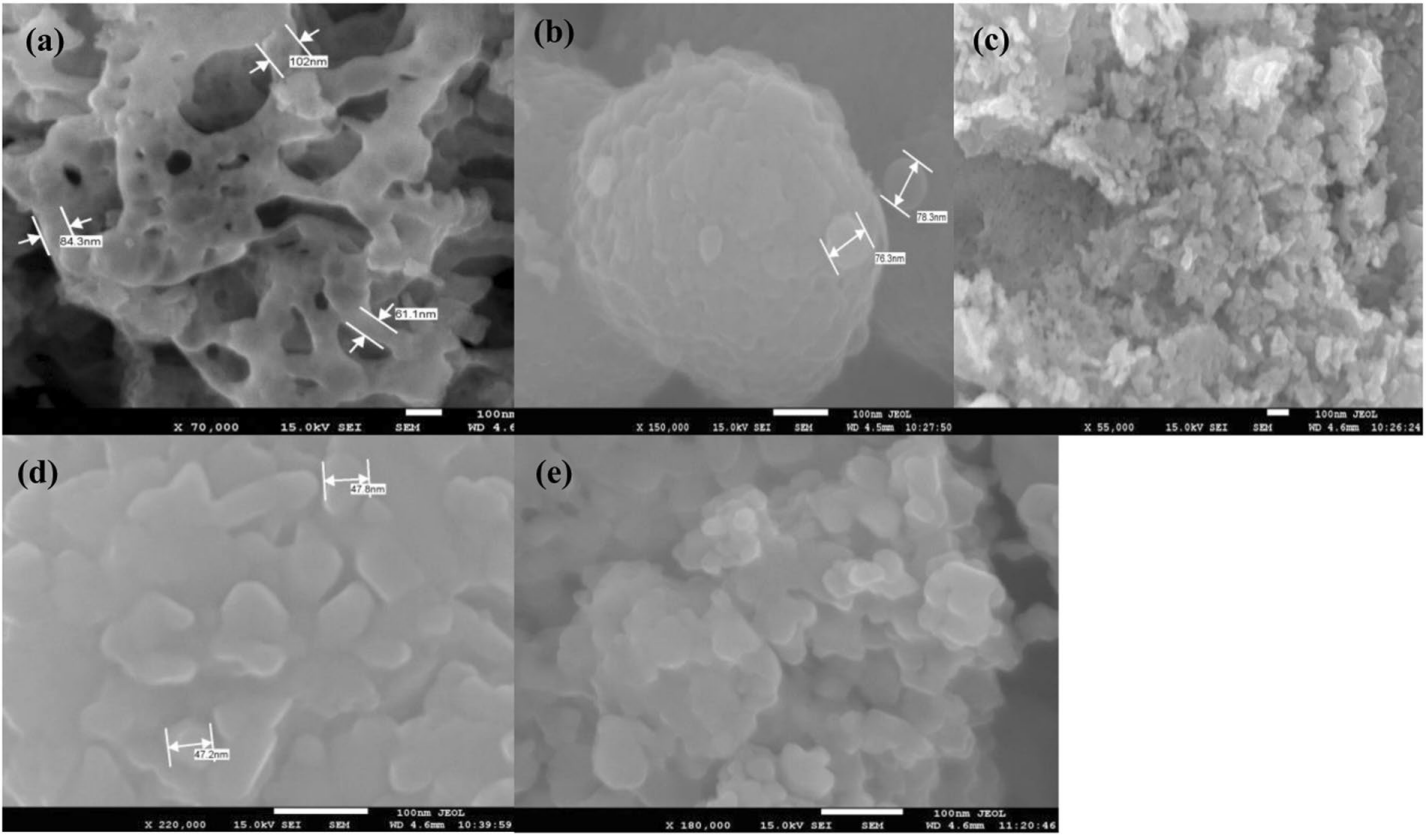
SEM micrographs of NdMnO_3_ (**a**), Nd0_.7_Sr_0.3_MnO_3_ (**b**), Nd_0.5_Sr_0.5_MnO_3_ (**c**), Nd_0.3_Sr_0.7_MnO_3_ (**d**) and Nd_0.1_Sr_0.9_MnO_3_ (**e**) nanocomposites.

Since the material properties depend strongly on its morphology, microstructural features for all synthesized samples have been examined using field emission scanning electron microscopy (FESEM) at room temperature. The obtained FESEM images of Nd_1−x_Sr_x_MnO_3_ (x = 0.0–0.18) nanocomposites are shown in Fig. 5. The FE-SEM micrographs revealed that the microstructure in our case consists of very small, randomly oriented, homogenous, well-interlinked and non-uniform (in shape and size) grains. Also the micrographs show the agglomeration of nanoparticles and all the samples are formed homogenously. The crystalline size decrease with increasing the concentration of strontium up to x = 0.5 and when x more than 0.5 the crystalline size increases. That is meaning the crystalline size in minimum at x = 0.5.

### Optical properties

**Figure 6:**
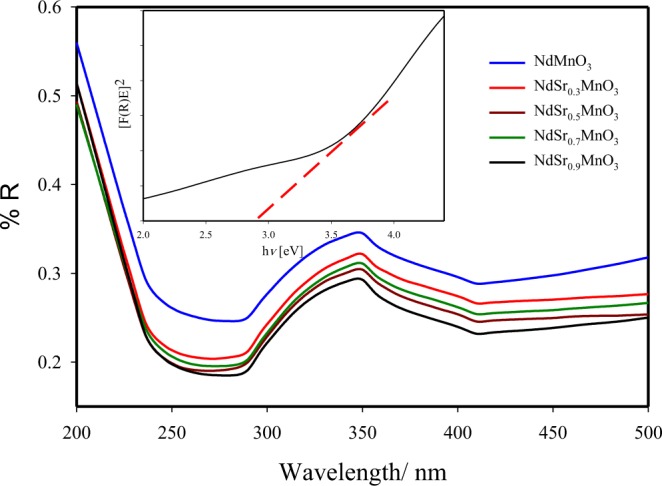
Diffuse reflectance UV-visible spectra of undoped NdMnO_3_ doped Nd_0.7_Sr_0.3_MnO_3_, Nd_0.5_Sr_0.5_MnO_3_, Nd_0.3_Sr_0.7_MnO_3_ and Nd_0.1_Sr_0.9_MnO_3_ nanocomposites; Inset Plot of transferred Kubelka–Munk versus energy of the light absorbed of Nd_0.3_Sr_0.7_MnO_3_ nanocomposite.

The optical properties of the undoped NdMnO_3_ and doped Nd_0.7_Sr_0.3_MnO_3_, Nd_0.5_Sr_0.5_MnO_3_, Nd_0.3_Sr_0.7_MnO_3_ and Nd_0.1_Sr_0.9_MnO_3_ nanocomposites were examined by the UV-vis diffuse reflectance spectroscopy (Fig. 6). This result indicated that Nd_0.7_Sr_0.3_MnO_3_ can harvest solar energy. After doping of Sr nanoparticles, the edge of optical absorption band was blue-shifted (Fig. 6). The spectral visible light absorbance range was increased with increasing of the Sr nanoparticles content. The bandgap of undoped NdMnO_3_ and Nd_0.7_Sr_0.3_MnO_3_, Nd_0.5_Sr_0.5_MnO_3_, Nd_0.3_Sr_0.7_MnO_3_ and Nd_0.1_Sr_0.9_MnO_3_ nanocomposites was estimated to be 3.05, 3.04, 3.02, 2.92 and 3.02 eV (Fig. 6, inset), respectively. It is clearly seen that the optical direct bandgap showed a slight shift to lower energies in the Nd_0.3_Sr_0.7_MnO_3_ nanocomposites from 3.05 eV to 2.92 with doping 0.7 Sr as a result of the interaction between Sr and undoped NdMnO_3_.

### Photocatalytic properties

**Figure 7:**
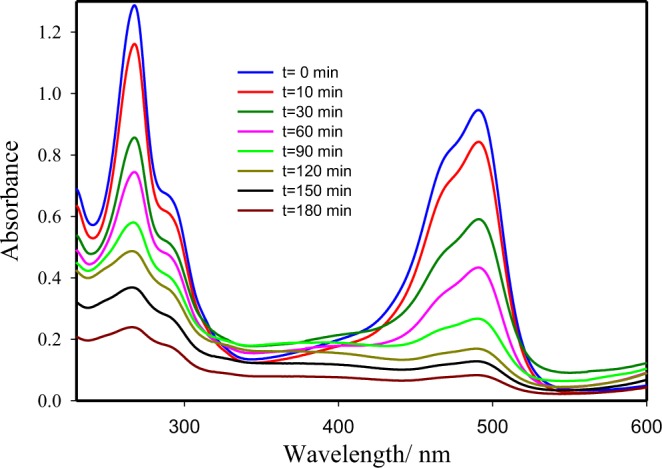
Absorbance vs. wavelength as a function of illumination time for the photocatalytic degradation of AO dye using Nd_0.3_Sr_0.7_MnO_3_ nanocomposites.

**Figure 8:**
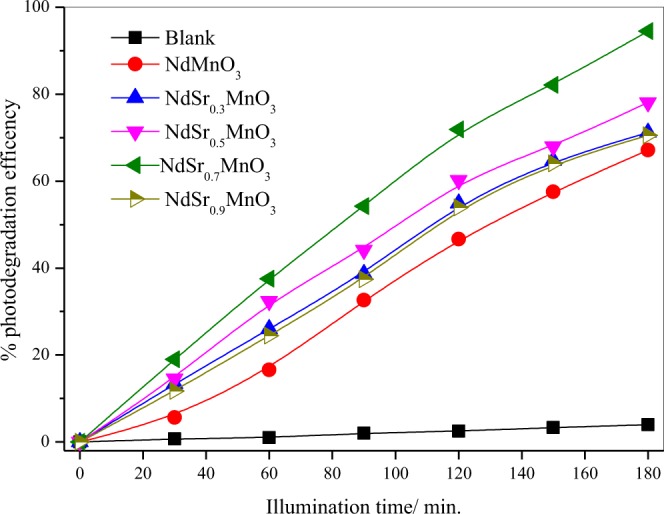
Photodegradation efficiency of AO under visible light irradiation as a function of illumination time in the presence of NdMnO_3_, Nd_0.7_Sr_0.3_MnO_3_, Nd_0.5_Sr_0.5_MnO_3_, Nd_0.3_Sr_0.7_MnO_3_ and Nd_0.1_Sr_0.9_MnO_3_. (experimental conditions: C_o_ = 3 × 10^−5^ molL^−1^; catalyst dose = 0.5 g L^−1^; temperature = 25 °C).

The photocatalytic performance of the NdMnO_3_, Nd_0.7_Sr_0.3_MnO_3_, Nd_0.5_Sr_0.5_MnO_3_, Nd_0.3_Sr_0.7_MnO_3_ and Nd_0.1_Sr_0.9_MnO_3_ nanocomposites for photodegradation of Acridine orange dye (AO) was evaluated over visible light illumination. The photocatalytic degradation of AO [3.0 × 10^–5^ molL^−1^] aqueous solution containing 0.5 gL^−1^ photocatalyst is depicted in Figs 7, 8. The absorption of AO in the UVA-vis region of the solar spectrum was determined to be substantially dependent on the Sr contents in Nd_1−x_Sr_x_MnO_3_ photocatalysts. The intense absorption peaks of AO determined at λ = 267 nm and λ = 490 nm gradually reduce by boosting illumination times. This experiment obviously exhibits that the AO decoloration can be completed throughout visible light illumination. The absorbance was reduced from 0.98 to 0.05 after nearly 3 h of illumination time (Fig. 7). The change in the AO dye concentration was depicted as a function of the illumination time. There was insignificant decrease in AO dye concentration was observed in the dark without a light source and also by illumination in the absence of Sr doped NdMnO_3_ photocatalyst, It is clearly seen that 95% of the initial AO dye concentration was degraded after 3 h illumination time (Fig. 8).

**Figure 9:**
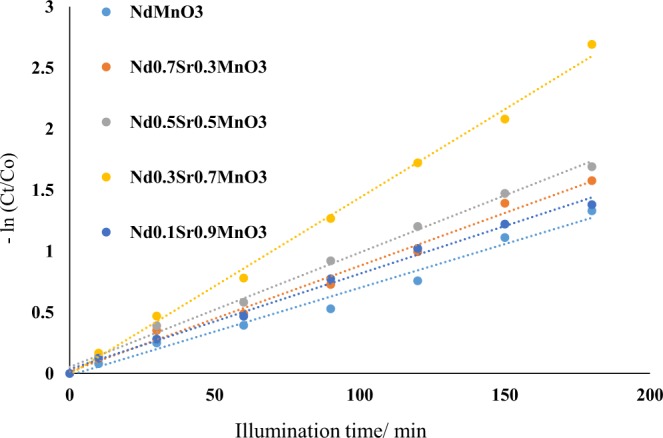
Pseudo first-order kinetic model plots in the presence of NdMnO_3_, Nd_0.7_Sr_0.3_MnO_3_, Nd_0.5_Sr_0.5_MnO_3_, Nd_0.3_Sr_0.7_MnO_3_ and Nd_0.1_Sr_0.9_MnO_3_ photocatalysts.

Figure 8 shows the pseudo first-order kinetic model plots in the presence of NdMnO_3_, Nd_0.7_Sr_0.3_MnO_3_, Nd_0.5_Sr_0.5_MnO_3_, Nd_0.3_Sr_0.7_MnO_3_ and Nd_0.1_Sr_0.9_MnO_3_ photocatalysts. This is justified in terms of the Langmuir-Hinshelwood model modified to harmonize taking place at interface of solid and liquid reactions^50–52^. The photodegradation of AO dye in aqueous solution was examined quantitatively using fitting the obtained experimental data to the model of Langmuir-Hinshelwood^53^.

$${\rm{R}}=(\frac{-dC}{dt})={{\rm{k}}}_{{\rm{r}}}\,\Theta =(\frac{krKC}{1+KC})$$

where, R is the reaction rate, C is the reactant concentration, k_r_ is the reaction rate constant, K is the adsorption coefficient of the reactant, and θ is the surface coverage. The product KC is insignificant with respect to unity^54^, and the above equation can be derived to the following pseudo-first order rate^55^:

$$-{\rm{In}}(\frac{Ct}{Co})={{\rm{k}}}_{{\rm{app}}}\,{\rm{t}}$$

where C_t_ and C_o_ are the reactant concentrations at times t and 0, respectively, and k_app_ (min^−1^) is the apparent reaction rate constant calculated by plotting -ln(C_t_/C_o_) versus the reaction time (t). Figure 9 exhibits the superb linearity between −ln(C_t_/C_o_) and t that the photodegradation of AO dye can be rationally determined by the pseudo-first order rate model. The calculated k_app_ are amounted to be 7.16 × 10^−3^, 7.80 × 10^−3^, 9.36 × 10^−3^, 14.47 × 10^−3^ min^−1^ and 8.65 × 10^−3^ min^−1^ for NdMnO_3_, Nd_0.7_Sr_0.3_MnO_3_, Nd_0.5_Sr_0.5_MnO_3_, Nd_0.3_Sr_0.7_MnO_3_ and Nd_0.1_Sr_0.9_MnO_3_, respectively and are in agreement with their photodegradation efficiencies (Fig. 9). These findings shows convincingly that the Nd_1−x_Sr_x_MnO_3_ photocatalysts possess great promise for visible light driven photodegradation of AO dye and the apparent reaction rate constant of Nd_0.3_Sr_0.7_MnO_3_ is greater 2 times than that undoped NdMnO_3_ nanocomposites.

**Figure 10:**
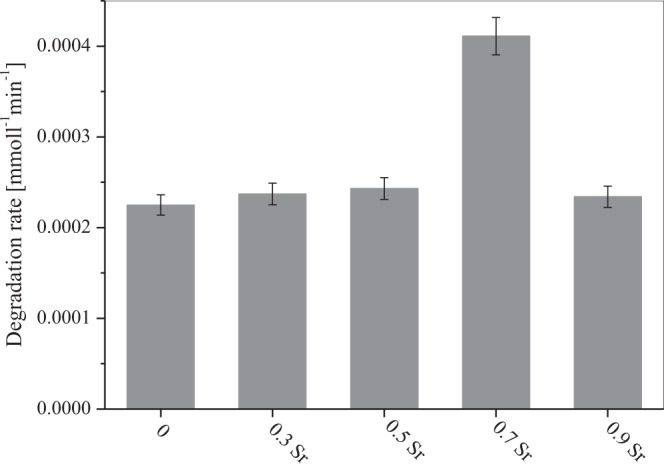
Comparison of photodegradation rate of NdMnO_3_, Nd_0.7_Sr_0.3_MnO_3_, Nd_0.5_Sr_0.5_MnO_3_, Nd_0.3_Sr_0.7_MnO_3_ and Nd_0.1_Sr_0.9_MnO_3_ for the decomposition of AO dye.

In particular, Nd_0.3_Sr_0.7_MnO_3_ exhibits the highest rate constant, which can be explained to its largest surface area (22–30 m^2^/g). The high surface area of Nd_0.3_Sr_0.7_MnO_3_ produces much more active sites, which in turn become higher absorption of visible light energy and substantially boosts the photodegradation performance. On the other hand, the AO photodegradation rates in the presence of NdMnO_3_, Nd_0.7_Sr_0.3_MnO_3_, Nd_0.5_Sr_0.5_MnO_3_, Nd_0.3_Sr_0.7_MnO_3_ and Nd_0.1_Sr_0.9_MnO_3_ nanocomposites are shown in Fig. 10. The results revealed that the photodegradation rate of AO goes much more speedily in the presence of Nd_0.3_Sr_0.7_MnO_3_ nanocomposite [4.11 × 10^−7^mol L^−1^ min^−1^] as compared to undoped NdMnO_3_ [2.25 × 10^−7^mol L^−1^ min^−1^]. It was demonstrated that the photodegradation rate was found to increase linearly with increasing Sr contents from 0.3 to 0.7wt % and decrease thereafter. Interestingly, the Nd_0.3_Sr_0.7_MnO_3_ content has the highest degradation rate of AO which is two times faster than undoped NdMnO_3_. This superior behavior in photocatalytic activity of Nd_0.3_Sr_0.7_MnO_3_ nanocomposite emerges from large surface area, structural anisotropy, and small particle size^56^. As shown in XRD findings, the phase crystalline NdMnO_3_ nanocomposite is monoclinic and orthorhombic crystals. At Sr content x = 0.3 and x = 0.5, the monoclinic phase is completely transformed into the orthorhombic crystal structure form. With increasing of the Sr content >0.5 a new phase appeared (monoclinic with space group P21/n) along with the orthorhombic Pbnm phase. Li *et al*.^57^ explained that sodium niobate can display anisotropy (i.e., the band gap calculated from absorption studies was different along different planes) in its photocatalytic activity and OH^−^ ion generation. They implied that anisotropy in photocatalytic activity could be resulted from some intrinsic properties such as anisotropy in ferroelectric properties. Moreover, the wide band gap of sodium niobate may make it less photoresponsive in the UV range of spectrum. Thus, we need for a range of strategies, like doping and sensitizing the photocatalyst with a narrow-band-gap semiconductor to improve the efficiency in the visible region of the solar spectrum of the sodium niobate.

**Figure 11:**
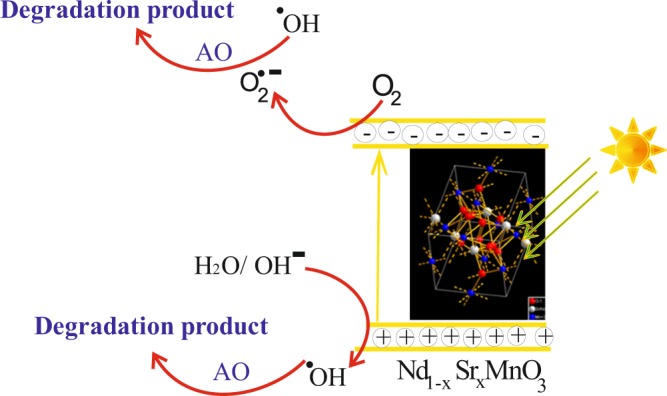
A plausible mechanism to give interpretation of the degradation of Acridine orange dye (AO) over Nd_1−x_Sr_x_MnO_3_ photocatalysts under visible light irradiation.

A plausible mechanism to give interpretation of the degradation of AO by Nd_1−x_Sr_x_MnO_3_ photocatalysts under visible light irradiation was proposed in our case as shown in Fig. 11. The photocatalytic activity mechanism of Nd_0.3_Sr_0.7_MnO_3_ is explained as follows: Upon illumination, the charge carriers were generated and the electrons were excited from valence and to conduction band. At the conduction band, the photogenerated electrons were reduced the adsorbed molecular O_2_ to produce O_2_^−•^. The O_2_^−•^ then reacts with H^+^ to form H_2_O_2_, which in turn is rapidly decomposed to ^•^OH. Finally, both ^•^OH and O_2_^−•^, being very strong oxidizing agents, remarkably raises the oxidation of AO dye into CO_2_, H_2_O, mineral acids, etc., and thus efficiently promote the overall photocatalytic efficiency^58^.

## Conclusions

Nd_1−x_Sr_x_MnO_3_ nanocomposites perovskites at different Sr doping content (0.3, 0.5, 0.7 and 0.9) were synthesized using sol gel method. XRD fingings showed that the sample without strontium (x = 0) is possesses two phases; monoclinic crystal system with space group C2/c and orthorhombic crystal system of space group Pbnm. As a result of adding strontium the monoclinic phase is completely transformed into the orthorhombic crystal structure at x = 0.3 and x = 0.5. With increasing strontium concentration more than 0.5, a new phase structures monoclinic with space group P21/n along with the orthorhombic Pbnm were observed. The optical direct bandgap showed a slight shift to lower energies in the Nd_0.3_Sr_0.7_MnO_3_ nanocomposites from 3.05 eV to 2.92 with doping 0.7 Sr as a result of the interaction between Sr and undoped NdMnO_3_. 95% of the initial AO dye concentration was degraded after 3 h illumination time. These findings shows convincingly that the Nd_1−x_Sr_x_MnO_3_ photocatalysts possess great promise for visible light driven photodegradation of AO dye and the apparent reaction rate constant of Nd_0.3_Sr_0.7_MnO_3_ is greater 2 times than that undoped NdMnO_3_ nanocomposites.
